# Human breast cancer and lymph node metastases express Gb3 and can be targeted by STxB-vectorized chemotherapeutic compounds

**DOI:** 10.1186/1471-2407-14-916

**Published:** 2014-12-04

**Authors:** Lev Stimmer, Sabrina Dehay, Fariba Nemati, Gerald Massonnet, Sophie Richon, Didier Decaudin, Jerzy Klijanienko, Ludger Johannes

**Affiliations:** Endocytic Trafficking and Therapeutic Delivery Group, UMR3666 CNRS — U1143 INSERM, Institut Curie—Centre de Recherche, 26 rue d’Ulm, 75248 Paris Cedex 05, France; CNRS UMR3666, 75005 Paris, France; U1143 INSERM, 75005 Paris, France; Laboratory of Preclinical Investigation, Translational Research Department, Institut Curie, Paris, France; Department of Tumor Biology, Institut Curie, Paris, France; Faculté des Sciences Pharmaceutiques et Biologiques, CNRS - IMTCE - IFR71, 4, Avenue de l’Observatoire, 75006 Paris, France

**Keywords:** Breast cancer, ESL, Gb3, Shiga toxin, STxB

## Abstract

**Background:**

The B-subunit of Shiga toxin (STxB) specifically binds to the glycosphingolipid Gb3 that is highly expressed on a number of human tumors and has been shown to target tumor cells in mouse models and *ex vivo* on primary colon carcinoma specimen.

**Methods:**

Using a novel *ex vivo* STxB labeling (ESL) method we studied Gb3 expression in cytological specimens of primary human breast tumors from 107 patients, and in synchronous lymph node metastases from 20 patients. Fluorescent STxB was incubated with fine-needle aspiration (FNA) specimens, and Gb3 expression was evaluated by fluorescence microscopy. Furthermore, 11 patient-derived human breast cancer xenografts (HBCx) were evaluated for expression of Gb3 by ESL and FACS. In addition, the biodistribution of fluorescent STxB conjugate was studied after intravenous injection in a Gb3 positive HBCx model.

**Results:**

Gb3 expression was detected in 62 of 107 patients (57.9%), mainly in epithelial tumor cells. Gb3 positivity correlated with estrogen receptor expression (p ≤ 0.01), whereas absence of Gb3 expression in primary tumors was correlated with the presence of lymph node metastases (p ≤ 0.03). 65% of lymph node metastases were Gb3 positive and in 40% of tested patients, we observed a statistically significant increase of metastatic Gb3 expression (p ≤ 0.04). Using concordant ESL and flow cytometry analysis, 6 out of 11 HBCx samples were scored positive. Intravenous injections of fluorescent STxB into HBC xenografted mice showed preferential STxB accumulation in epithelial cells and cells with endothelial morphology of the tumor.

**Conclusion:**

The enhanced expression of Gb3 in primary breast carcinomas and its lymph node metastases indicate that the development of STxB-based therapeutic strategies is of interest in this pathology. Gb3 expressing HBCx can be used as a model for preclinical studies with STxB conjugates. Finally, the ESL technique on FNA represents a rapid and cost effective method for the stratification of patients in future clinical trials.

## Background

Breast cancer is the most common malignancy affecting women in the Western world. Besides surgery, radiation, chemotherapy, and endocrine treatment, immunotherapy has become an established part of systemic therapy in treating metastatic breast cancer [1]. Several single-agent and combination chemotherapeutic options have been shown to be effective as first- or second-line therapy in the management of metastatic disease with platinum derivates and anthracyclines being the most active compounds [2]. These often have substantial side effects, however, and the identification of new agents for highly specific targeted therapy is an important area of cancer research. Bacterial toxins such as adenylate cyclase, botulinum, cholera, and Shiga-like toxins (verotoxins) might be used to establish novel therapeutics against tumor malignancies, either as independent anti-neoplastic agents or in combination with chemo- or radiotherapy. Their capacity to target specific signaling pathways might reduce side-effects [3].

Shiga and Shiga-like toxins are produced by *Shigella dysenteriae* and enterohemorrhagic strains of *Escherichia coli*. These toxins are composed of two non-covalently attached parts: the enzymatically active A-subunit, and the non-toxic, pentameric B-subunit (STxB) [4]. STxB specifically binds to the sugar moiety of the glycosphingolipid globotriaosylceramide (known as CD77, Gb3, and ceramide trihexoside) in the plasma membrane of target cells, and mediates uptake and intracellular transport of the toxin [5, 6]. Shiga toxin is internalized by clathrin-independent endocytosis [7], and is then transported to the endoplasmic retriculum following the retrograde route [8]. The A-subunit is cleaved in the *trans*-Golgi network, and the enzymatically active A1 part is translocated from the lumen of the endoplasmic reticulum to the cytosol. The A1 fragment irreversibly modifies ribosomal 28S RNA, leading to the inhibition of protein biosynthesis and cell death by apoptosis [5].

Deregulation of Gb3 expression has been described in different human and animal malignancies [9]. Furthermore, increased expression of Gb3 was reported for several solid tumors such as breast [10], ovarian [11], pancreatic [12, 13] and colon [13, 14] cancers, and malignant meningioma [15]. Tumor-associated Gb3 is accessible to natural ligands (i.e. Shiga-like toxins), making them candidates for oncological applications [5]. The anti-neoplasic activity of Shiga toxin has been documented in xenograft models of astrocytoma, renal cancer and malignant meningioma [15–18]. However, the use of holotoxin in humans might be problematic since the action of the catalytic A-subunit is not tumor cell specific and is likely to cause kidney damage. In contrast, STxB might be developed into a delivery tool for therapeutic entities that by themselves have some tumor specificity.

Further development of STxB-based targeted therapies of human breast cancer requires exact knowledge about Gb3 expression in primary tumors and lymph node metastases. Furthermore, the development of rapid and cost effective diagnosis methods is essential for the appropriate choice of pre-clinical models and for the stratification of patients in future clinical trials. The purpose of this study was to evaluate the expression of Gb3 in the cytological specimens of primary and metastatic human breast carcinoma, as well as in HBCx, using a novel *ex vivo* STxB labeling (ESL) technique.

Here, we report that the majority of breast cancer patients express Gb3 in primary tumors, and increase Gb3 expression in lymph node metastases in 40% of the cases. Furthermore, a novel ESL technique was developed as a useful tool for the detection of Gb3 expression in tumor cells. Finally, STxB conjugate accumulates in Gb3 positive HBCx after intravenous injection, indicating that STxB-based therapeutic strategies might be of interest in this pathology.

## Methods

### Breast cancer patients

All used human cytological or histological samples were residuals of specimens sampled during conventional medical consultation. The Institutional Review Board of *Institute Curie* approved the study. The Institutional approval was elaborated according to Helsinki's Declaration of human rights. Human breast cancer specimens were obtained following informed consent from all patients undergoing cytological or histological examination.

Tumor specimens were collected between January 2009 and January 2012 from 87 patients with previously untreated primary breast carcinomas. Furthermore, 20 patients with previously untreated breast carcinomas associated to clinically palpable axillar lymph node metastases were available for this study. Clinicopathological patient data (i.e. size of primary tumor, location, lymph node extension) were recorded. Additional samples, including 20 samples of invasive breast carcinoma, 12 samples of mammary adenofibroma, 14 samples of normal breast tissue, and 7 healthy kidney samples were available for Gb3 extraction.

Primary lesions and lymph node metastases were fine-needle sampled. For diagnosis, a large part of aspirated material was smeared onto slides and stained according to the May-Grünwald-Giemsa (MGG) method. The remainder of the cytological material was placed in Dulbecco's Modified Eagle Medium (DMEM) without supplements for the *ex vivo* STxB labeling (ESL) procedure. Immediately after aspiration, a histological biopsy was also performed after local anesthesia. Patients were treated by neoadjuvant chemotherapy according to the Institute’s guidelines, and finally underwent surgery at the primary site with axillary lymph node cleaning. Biopsies from primary lesions were immediately fixed and stained with hematoxylin-eosin-safran. Histological sections were evaluated according to Elston-Ellis histological grading including a mitotic count on 10 high power fields. Estrogen and progesterone receptors as well as HER2 expression were evaluated using a set of monoclonal antibodies: ER (clone 6F11; 1/200; Novocastra, Rungis, France), PR (clone 1A6; 1/200; Novocastra), and HER2 (clone CB11; 1/1,000; Novocastra). Proliferation index was assessed using monoclonal anti-Ki67 antibody (Ki67, clone MIB-1; 1/75; DAKO, France). All primary tumors were classified according St. Gallen International Expert Consensus of 2011 [19]. This classification distinguishes following categories: Luminal A (ER and/or PR positive, HER2 negative and Ki67 low), Luminal B/HER2- (ER and/or PR positive, HER2 negative and Ki67 high), Luminal B/HER2+ (ER and/or PR positive, HER2 positive), HER2+ (ER and PR negative, HER2 positive), Triple Negative (ER, PR, HER2 negative).

### Patient-derived human breast cancer xenograft model

Tumor specimens were obtained from breast cancer patients and established as xenografts, as previously described [20]. Briefly, fresh tumor fragments were grafted subcutaneously into the interscapular fat pad of female Swiss nude mice under anesthesia. Mice were kept in specific pathogen-free animal housing at *Institut Curie*, and received estrogen (17 mg/mL) diluted in drinking water. HBCx appeared at the graft site two months after grafting. These were subsequently transplanted from mouse to mouse. The experimental protocol and animal housing were in accordance with institutional guidelines as proposed by the French Ethics Committee (Agreement B75-05-18). 11 HBCx established from different patients were available for cytological and flow cytometry analysis using STxB-Cy3 and STxB-Alexa Fluor® 488 conjugates, respectively. The recombinant mutant STxB-Cys was produced in our laboratory as previously described [21] (see below). Fine-needle aspiration followed by ESL was performed after the xenografts reached 10 to 12 mm of diameter. The animals were sacrificed, xenograft were removed and placed in sterile DMEM solution at 4°C till flow cytometry analysis. Furthermore, ER, PR and HER2 status of all HBCx was evaluated by immunohistochemistry under the same conditions as described above.

### Purification of STxB

Recombinant STxB was purified from bacteria as previously described [21]. Briefly, bacterial extracts were loaded on a Q Sepharose High Performance strong anion exchange column (GE Healthcare) and eluted in a linear NaCl gradient (25 mM Bis-Tris–HCl, pH 6). STxB eluted from the column at around 150–250 mM, and was dialyzed against coupling buffer (20 mM HEPES-KOH, pH 7.4, 150 mM NaCl), and subjected to coupling with Cy3 (cyanine 3; Amersham Biosciences) or Alexa 488 (AlexaFluor® 488, Life Technologies) fluorochromes according to the supplier's instructions. Cy3- and Alexa 488-coupled STxB were purified by PD-10 column (GE Healthcare), snap frozen and stored at -80°C.

### Ex vivo STxB Labeling (ESL) method

This technique was applied to the fine needle aspirates obtained from patients and from xenografted mice. One part of aspirated material was smeared on slides and stained according to the May-Grünwald-Giemsa method for estimation of cellularity. Another part was placed in Dulbecco's Modified Eagle Medium (DMEM) for ESL. The isolation of cancer cells required centrifugation through a density gradient. 3 ml of cell suspension was layered onto a sterile aqueous medium containing ficoll and sodium diatrizoate at a predetermined density of 1.199 and 1.077 g/ml at 25°C. Centrifugation at 1800 rpm for 20 min at room temperature resulted in the separation of epithelial and white blood cells that accumulated at the interface of the two ficoll fractions, from red blood cells that passed through the interface and formed a pellet at the bottom of the tube. The cancer cell-fraction was collected and washed with sterile DMEM to remove contaminating separation medium. After the last centrifugation step cells were suspended in 1 ml of DMEM containing 1 μM of STxB-Cy3, and incubated for 1 hour at 25°C with slow agitation. After two wash steps in phosphate buffered saline, 150 μl of cell suspension was projected onto slides by centrifugation at 500 rpm for 8 min using a Shandon CytoSpin 2 Cytocentrifuge. Slides were air dried, and nuclei labeled with DAPI. To confirm the epithelial nature of extracted cells, labeling with anti-AE1/AE3 antibody (Dako, polyclonal, rabbit anti human, 1/200) was performed. Samples were observed by epifluorescence microscopy and evaluated for the presence of STxB-Cy3 labeling. Samples were judged suitable for this study when at least 100 cells with epithelial morphology were counted on the slide. Patients with lower cell count were excluded from the study. Cell density and morphology were judged on MGG stained slides and on ESL slides using DAPI nuclear staining.

### Gb3 extraction from normal tissue, breast cancer specimens and HBCx

Tumors and healthy breast tissues as well as breast cancer xenografts were frozen in liquid nitrogen immediately after surgery, and stored at -80°C. Tissues were weighed and mechanically homogenized in 1 mL of water. Gb3 expression was quantified after lipid extraction, STxB overlay, and immunodetection as previously described [22]. Briefly, lipids were extracted using chlorophorm/methanol (1:2), followed by drying of the chloroform phase, and saponification of lipids at 56°C for 1 hour in 1 ml of methanol/KOH. The isolated neutral glycolipids were separated on high-performance thin-layer chromatography (TLC) plates (Merck, Darmstadt, Germany), and visualized by incubation with STxB (20 nM) and subsequent immunolabeling.

### Flow cytometry of breast cancer xenografts

Single-cell suspensions were prepared from excised tumors as previously described [23]. Briefly, tumors were removed from sacrificed mice, minced and incubated twice in non-enzymatic dissociation buffer (Invitrogen) followed by mild enzyme digestion step including collagenase III (200 U/ml; Sigma-Aldrich, St Louis, MO, USA), DNase I (200 U/ml; Sigma-Aldrich), each incubation for 30 min at 37°C. Between each step the suspensions were filtered through a 40 μm nylon mesh cell strainer (BD Biosciences, San Diego, CA, USA), and released cells were centrifuged at 1200 rpm for 2 min, separated using a double Ficoll gradient (Histopaque; Sigma-Aldrich; densities 1.077 and 1.119), washed in PBS, and stored at 4°C until staining and flow cytometry. Immunostaining was performed on all HBCx using Alexa Fluor® 488 labeled STxB (1 μM), mouse anti human EpCAM_PerCP-Cy5.5 (Biolegend), and rat anti mouse pan-H2_PE (Biolegend). Dissociated cells were incubated with antibody mix for 20 min at 4°C in the dark, and after washing, 2 ng/ml 40,60-diamidino-2-phenylindole (DAPI) (Invitrogen) was added. Data were acquired on a standard LSRII (BD Biosciences) with 488 nm laser excitation. ‘Fluorescence minus one’ (FMO) controls were used to establish negative cut-offs.

Live (negative for DAPI staining) single (by eliminating doublets in FSC-W/FSC-A scattergramms) human cells (EpCAM pos and panH-2 neg) were analyzed and results were calculated in ratio mean fluorescence intensity (MFI test/MFI FMO). Cell populations with ratio strictly over 1.5 were considered as positive for Gb3 expression. Data analysis was performed using the Flowjo software (Tree Star, Ashland, OR, USA).

### Intravenous injection of STxB-Cy3

To study the biodistribution of STxB-Cy3 conjugates *in vivo*, five mice were xenografted with the Gb3 expressing breast cancer HBCx-174, and injected with STxB-Cy3 (100 μg/20 g) in the retro-orbital sinus. Animals were sacrificed after 24 hours. One half of the xenograft was placed in DMEM for future FACS analysis, whereas another half as well as kidney, liver, spleen, lung, small and large intestine were sampled and fixed in 4% of PFA. Paraffin embedded sections were labeled with DAPI and evaluated by epifuorescence microscopy.

### Statistical analysis

Correlations between Gb3 expression and other variables were analyzed using Pearson's chi-square test. Additionally, Wilcoxon Signed Rank Test was carried out to compare the expression in primary tumor and lymph node metastases for each patient. Furthermore, one-way ANOVA was used to compare Gb3 expression in normal breast tissue, adenofibroma and breast carcinoma. The level of significance for rejection of the null hypothesis of no relationship between variables was taken to be p ≤ 0.05.

## Results

### Gb3 expression is increased in human breast carcinoma compared to normal tissue

**Figure 1:**
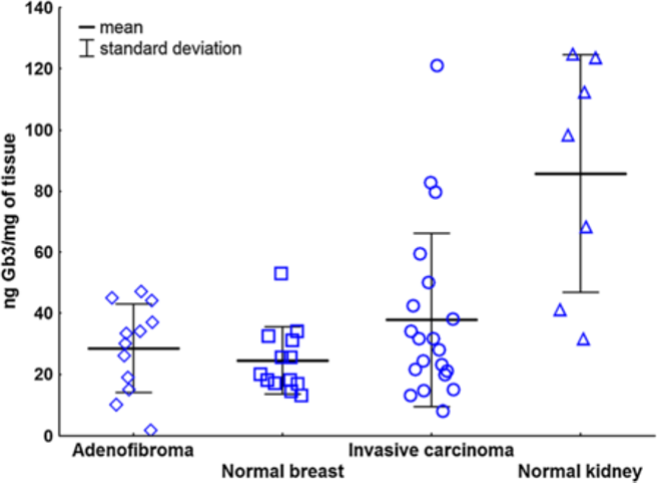
**Gb3 expression in normal breast tissue, adenofibroma, breast carcinoma and normal human kidney specimens.**

In order to evaluate global quantity of Gb3 in normal and tumor breast tissue, Gb3 was extracted and quantified using a previously published STxB overlay method [22] Normal breast tissue expressed Gb3 in the range from 13.0 to 53.0 ng per mg of tissue (mean 24.52 ng/mg), whereas breast carcinoma showed 1.5 times higher Gb3 expression (mean 37.9 ng/mg) with a higher range from 8.0 ng/mg to 121.0 ng/mg. However, this difference was not significant (p > 0.5). In contrast to breast carcinoma, adenofibroma showed comparable Gb3 levels to normal tissue (mean 28.51 ng/mg, min. 1.8 ng/mg, max. 45.0 ng/mg). In addition, normal kidney tissue, known for high Gb3 levels, was extracted as a positive control. This tissue showed mean values of 85.72 ng of Gb3 per mg of tissue (min. 31.5 ng/mg, max. 124.7 ng/mg), which was 2.3 times higher than the mean of Gb3 in breast carcinoma. These data are summarized in Figure 1.

### Gb3 positivity correlates with estrogen receptor expression in primary human breast cancer

**Figure 2:**
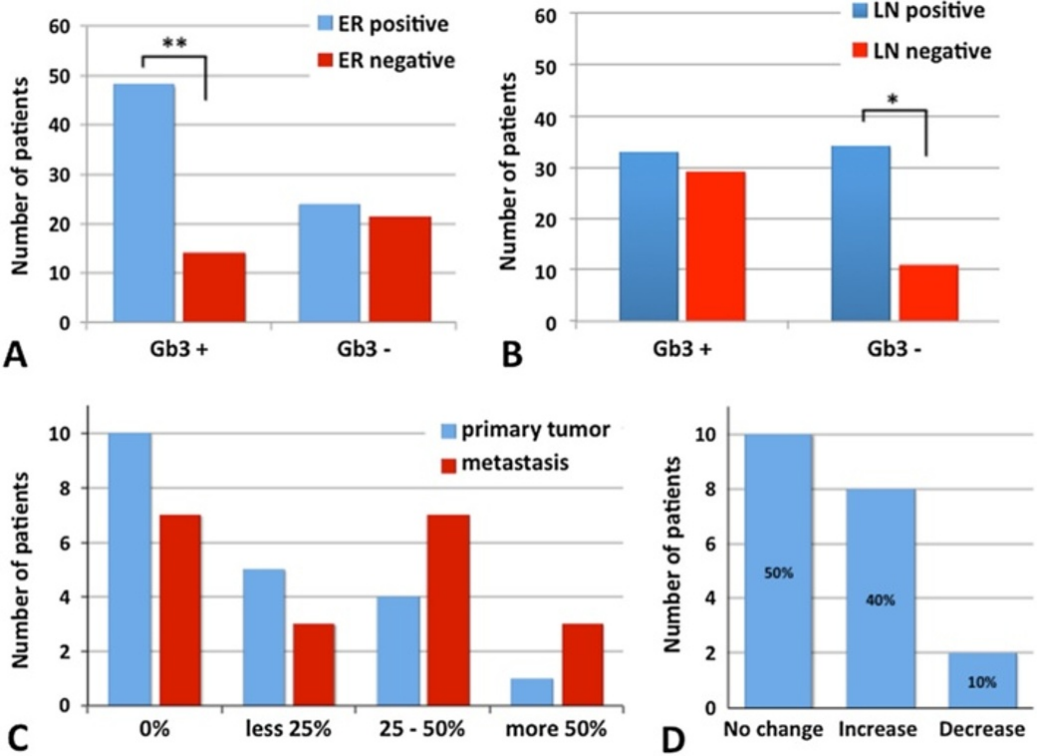
**Gb3 expression in primary breast tumors and lymph node metastases. A**: Estrogen receptor and Gb3 expression in primary breast tumors. Note: Gb3 positivity (Gb3 + group) is correlated with estrogen receptor expression. **: p ≤ 0.01, ER: estrogen receptor. **B**: Presence of axillary lymph node metastases in patients with and without Gb3 expression in primary breast cancer. Note a higher number of patients with lymph node metastases in the Gb3 negative group. *: p ≤ 0.03, LN: lymph node. **C**: Relative numbers of Gb3 expressing cells. **D**: Change in Gb3 expression between primary and metastatic tumors. Note: 40% of patients showed relative increase, whereas 50% had no change, and 10% lost Gb3 expression in the lymph node metastases compared to the primary tumor.

Fine-needle aspiration (FNA) is a standard cytological tool for diagnostics of suspected primary or secondary malignancies [24]. In order to evaluate Gb3 expression on epithelial cancer cells, we developed a novel technique for the *ex vivo* STxB labeling (ESL) in which live cells from dissociated tumors are incubated with STxB prior to microscopical viewing (see Methods section). We combined the FNA and ESL techniques on cytological specimens of primary breast cancers of 107 patients. Roughly 100 to 500 cells with epithelial morphology per patient were subjected to ESL. Cell density and morphology were judged on MGG stained slides and on ESL slides using DAPI nuclear stain. Examined patients were separated in two groups according to Gb3 expression in primary tumors. 62 patients (57.9%) expressed Gb3 in primary tumor (Gb3 positive group). Regarding the receptor status of Gb3 positive patients, 48/62 (77.4%) were positive for estrogen receptor (ER), 35/62 (56.5%) for progesterone receptor (PR), and 40/62 (64.5%) for HER2. 5/62 (8.1%) were triple negative (Table 1). Using Pearson’s chi-squared test, Gb3 expression was significantly correlated to estrogen receptor expression (p ≤ 0.01; Figure 2A), whereas no correlation was found with age, histological type, grade, molecular classification, mitotic index, progesterone receptor or HER2 expression. Furthermore, no significant relationship was found between Gb3 positivity and the presence of lymph node metastases. 33 of 62 Gb3 positive patients (53.2%) had lymphatic extension, whereas 29/62 patients (46.8%) had no lymph node metastases.Absence of Gb3 expression (Gb3 negative group) was observed in 45 patients (42.1%). Gb3 negativity was significantly correlated (p ≤ 0.03) with higher number of lymph node metastases in this group (Figure 2B); including 34/45 lymph node positive (75.6%) and 11/45 lymph node negative (24.4%) patients. No correlation was found in this group with age, histological type, grade, molecular classification, mitotic index, estrogen and progesterone receptor or HER2 expression.

**Table 1:** **Gb3 expression and clinicopathological characteristics of primary breast carcinomas**

Feature	Gb3 +	Gb3 -
**Patients**	62	45
Age**	51.0	53.9
**Lymph node status**		
Node-positive	33	34
Node-negative	29	11
**Histological type**		
Ductal	58	37
Lobular	1	3
Papillary	2	1
Others	1	4
Mitotic index*	2.0	2.5
Grade*	2.5	3.0
**Estrogen receptor**		
Positive	48	24
Negative	14	21
**Progesterone receptor**		
Positive	35	20
Negative	27	25
**HER2**		
Positive	40	25
Negative	22	20
**Molecular classification**		
Luminal A	6	7
Luminal B	42	16
Luminal B/HER2 +	31	15
Luminal B/HER2 -	11	1
HER2 positive	9	9
Triple Negative	5	10
Not classified	0	3

*Median of mitotic index and grade; **Mean.

In conclusion, Gb3 is expressed in the majority of primary breast carcinomas. Gb3 positivity correlates with estrogen receptor expression in primary breast cancer, whereas absence of Gb3 is linked to frequency of lymph node metastases.

### Gb3 expression is enhanced in lymph node metastases compared to the primary tumor in 40% of patients

Several receptors, including ER, PR and HER2 show discordant expression between primary tumor and lymph node metastases [25]. In order to assess Gb3 expression in primary and metastatic cancer we selected 20 patients with previously untreated breast carcinomas associated to clinical palpable pathological lymph nodes. Primary breast tumors and synchronous lymph node metastases were fine-needle sampled and examined by the ESL method. Gb3 expression was estimated in epithelial appearing cells using the following scale: low (<25% of labeled cells), moderate (25 - 50% of labeled cells) and high (>50% of labeled cells). Gb3 expression in primary breast tumors and lymph node metastases is summarized in Figure 2C. No expression was seen in 10 primary tumors and 7 metastases. Gb3 was expressed in 10 (50%) primary tumors, including low expression in 5, moderate in 4, and high in 1 patient. Furthermore 13 (65%) metastases showed STxB-Cy3 accumulation. This included low Gb3 expression in 3, moderate in 7, and high in 3 patients.We observed differences regarding the individual receptor expression between primary tumor and its lymph node metastases. 8 patients (40%) showed an increase of the relative Gb3 expression in lymph node metastases compared to primary tumor. This group included 5 patients showing no expression in primary tumor and low (2 patients) or moderate (3 patients) expression in lymph node metastases. Further two patients presented low number of positive cells in primary tumor and moderate (1 patient) or high (1 patient) expression in metastases. The remaining patient increased tumor Gb3 expression from moderate in mammary mass to high in axillary metastases. On the other hand, 10% (2 patients) showed a decreased expression from low level in primary tumor to no expression in nodal metastases. 10 patients (50%) had no change in the Gb3 expression, including 5 patients with no expression, 3 patients with moderate and 2 patients with low or high respective expression in both specimens (Figure 2D). Overall statistical analysis of connected probes of primary tumor and its lymph node metastases using Wilcoxon Signed Rank Test showed a significant increase in Gb3 expression in lymph node metastases in studied patients (p ≤ 0.04).

Our results show that Gb3 is expressed in 65% of lymph node metastases. Furthermore, 40% of patients showed an increase of the relative number of STxB positive cells between primary tumor and metastases.

### Gb3 is mainly expressed on tumor epithelial cells in FNA of primary and secondary breast cancers

**Figure 3:**
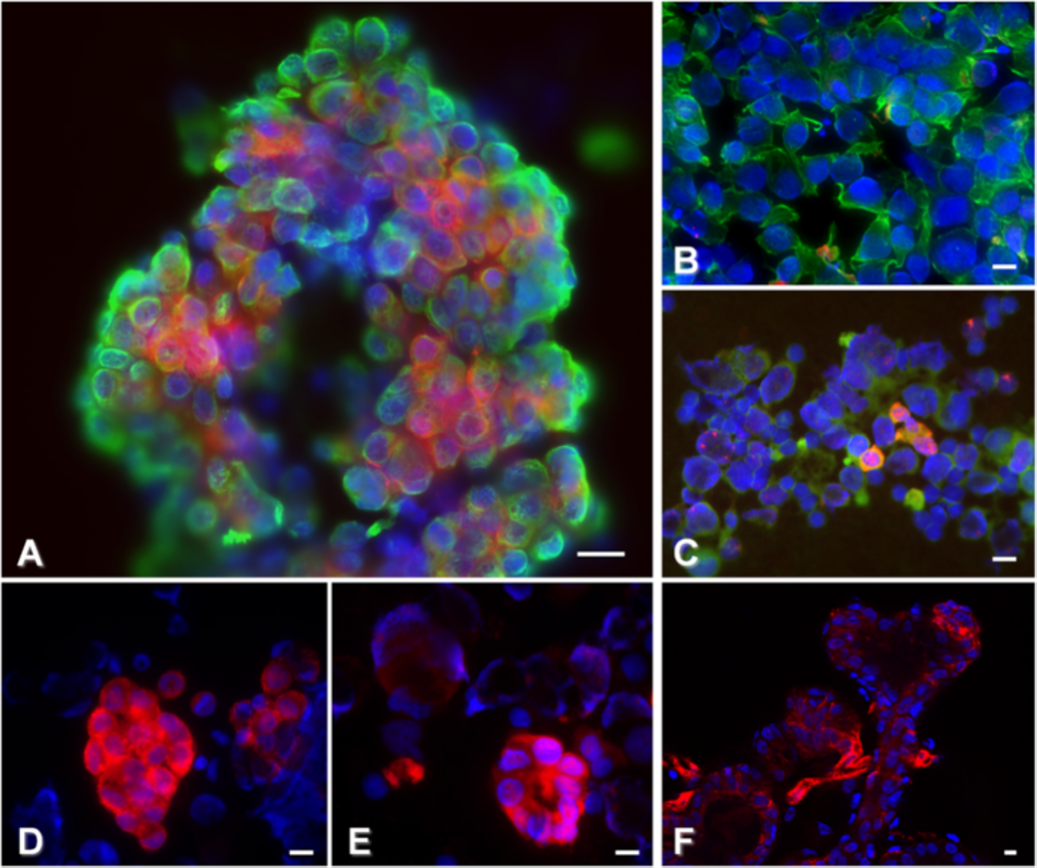
**Gb3 expression in fine-needle aspirates of human primary breast cancers and lymph node metastases. A**, **B**, **C**, **F**: Fine-needle aspirates from different patients. **A**: Tubular structure with Gb3 (red) expression within the cytokeratin expressing epithelial cells (green); **B**: isolated cytokeratin expressing epithelial cells (green) with no Gb3 expression; **C**: low number of Gb3 (red) expressing individualized epithelial cells (green); **F**: Gb3 (red) expression in cells with endothelial morphology forming a capillary-like structure (center). **D**, **E**: Gb3 expression (red) in lobular structures of a primary tumor **(D)**, and in tubular formations in lymph node metastases of the same patient **(E)**. Note similar morphological appearance, i.e. cohesive, polygonal cells forming lobular structures in both specimens. Bars: 20 μm.

In order to assess which cells express Gb3 in FNA of primary and secondary mammary cancers we used standard cytochemical MGG labeling as well as combined immunocytochemistry and ESL techniques. Roughly 100 to 500 cells with epithelial morphology were subjected to STxB accumulation. Patients with lower cell count were excluded from the study. Cell density and morphology was judged on MGG stained slides and on ESL slides using DAPI nuclear stain. The epithelial nature of STxB-positive cells was confirmed using immunocytochemical staining for pan-cytokeratins, showing a cytoplasmic co-localization of STxB-Cy3 and AE1/AE3 signals (Figure 3A). As shown in Figure 3A, D and E, labeling by STxB was noticed mainly in epithelial cells (i.e. larger epithelial tumor cells forming lobular, tubular and/or acinic structures). Isolated malignant cells were usually less positive than clustered cells (Figure 3B and C). The STxB signal was distributed mainly in the perinuclear region, or diffusely throughout cytoplasm, showing fine granular appearance. Furthermore, STxB staining was less frequently observed in neutrophils and rare in macrophages. Here, the signal was mostly cytoplasmic and appeared coarse granular. Structures with capillary morphology, which were sometimes seen, were strongly positive for STxB labeling (Figure 3F).

These results confirm that tumor epithelial cells are the major Gb3 expressing cell type in FNA of primary and metastatic breast cancer.

### Gb3 positive HBCx can be selected by ESL

**Figure 4:**
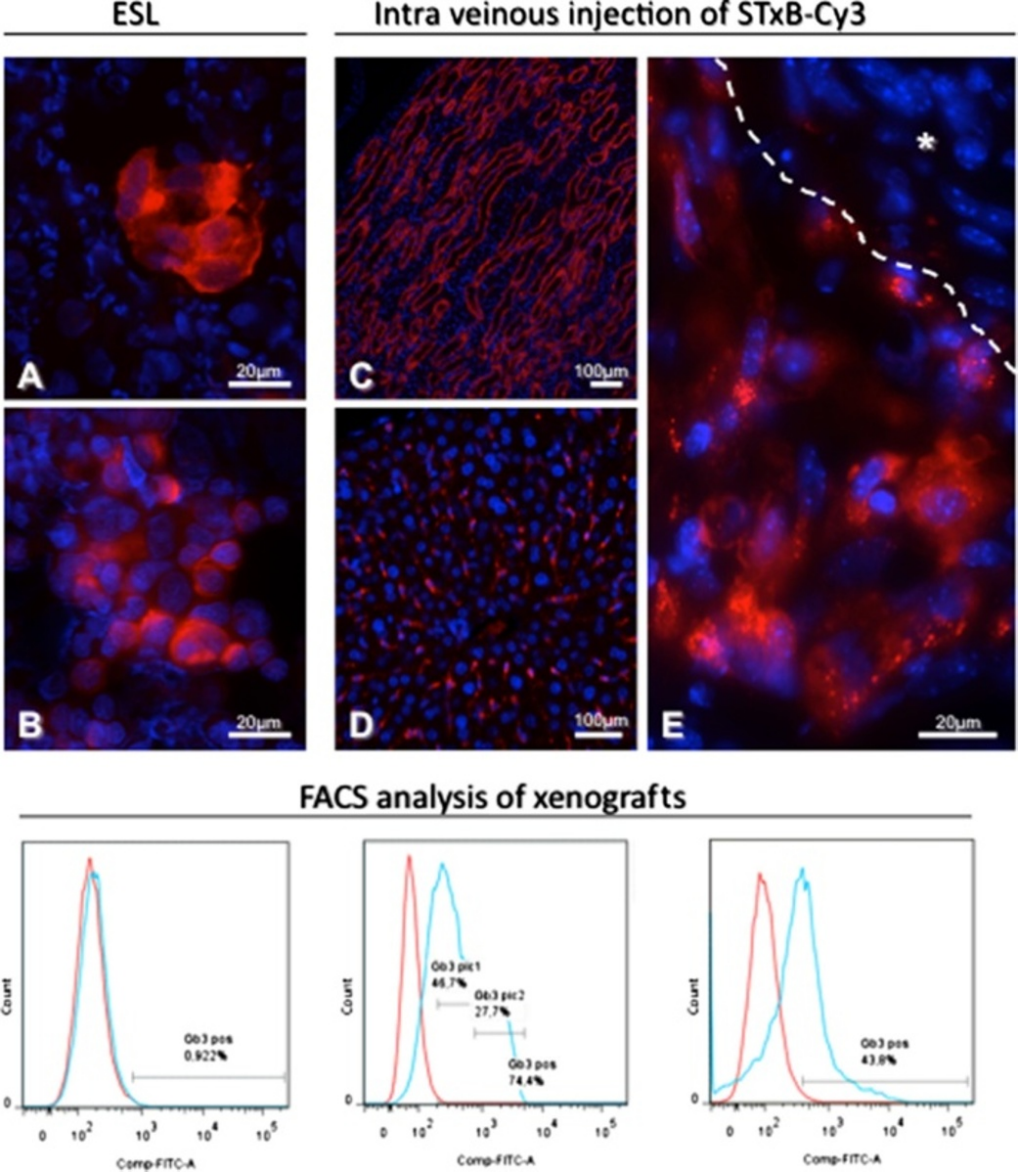
**Gb3 expression in mice with HBCx.** Upper part: STxB-Cy3 accumulation in tumor and organs after ESL or intravenous injection. **A**, **B**: Gb3 expression in fine-needle aspirates of HBCx with accumulation of STxB-Cy3 in tubular structures **(A)**, or individualized cells **(B)**. **C**: Diffuse and high expression of Gb3 in tubular structures of renal medulla, **D**: Diffuse and low expression in liver parenchyma in cells with endothelial morphology lining liver sinusoids, **E**: Gb3 expression in HBCx. Note a diffuse and high STxB-Cy3 accumulation in tumor cells with epithelial morphology compared to the absence of Gb3 expressing cells in tumor stroma (*). Lower part: FACS analysis of Gb3 expressing tumor cells in different HBCx. Note the lack of Gb3 positivity in HBCx-11 (left), the presence of two populations of Gb3 expressing cells in HBCx-52 (middle), and of one Gb3 positive population in HBCx-162 (right).

Breast cancer xenografts are useful tools for preclinical assessment of new treatments because of their similar molecular profile to the corresponding patient tumors [26]. Recent studies with these and similar breast cancer xenografts completed in our institution show that human breast cancer xenografts maintain the overall histologic, genomic and gene expression profile of the corresponding patient tumors and remains stable throughout sequential *in vivo* generations [20, 27]. ESL and FACS used for the evaluation of Gb3 expression in 11 HBCx. Concordant results between ESL and FACS were found in all tested HBCx: 6 were STxB-positive, and 5 negative (Table 2). Furthermore, using flow cytometry it was found that 4 out of 6 positive HBCx had two different tumor cell populations with high or low Gb3 expression levels. As illustrated in Figure 4A, Gb3 expressing cells were mainly morphologically well differentiated (i.e. cohesive polygonal epithelial cells forming lobular structures) and showed granular, often perinuclear intracytoplasmic labeling. Furthermore, less differentiated cells (i.e. groups of cells without particular formation) less frequently accumulated STxB (Figure 4B). In addition, 8 HBCx were available for Gb3 extraction. ESL and Gb3 extraction showed concordant results in 6 from 8 tested HBCx, including 4 positive and 2 negative xenografts. 2 HBCx were positive in Gb3 extraction and negative in ESL (Table 2). ER, PR and HER2 status was studied in all xenografts and the results are summarized in Table 2. Tested panel of HBCx included 1 ER+/PR-/HER2- and 2 HER2+ xenografts as well as 8 triple negative HBCx. There were no correlation between ER, PR or HER2 status and Gb3 expression.

**Table 2:** **ER, PR, HER2 and Gb3 expression in HBCx**

id	IHC			ESL	FACS						Gb3 extraction
	ER	PR	HER2	Gb3	n count	n pop	% min fluo	Ratio MFI	% max fluo	Ratio MFI	ng Gb3/mg of tissue
BC11	-	-	-	-	31499	1	100	1.5	x	x	N.A.
BC138	-	-	-	+	10579	2	85	1.2	15	13.1	2.7
BC143	-	-	-	-	9704	1	100	1.1	x	x	0.0
BC147	-	-	-	+	9051	2	85.6	2.9	14.7	22.9	45.7
BC151	-	-	+	-	2032	1	100	1.0	x	x	0.0
BC152	-	-	-	-	8444	1	100	1.0	x	x	15.0
BC162	-	-	-	+	25850	1	100	5.8	x	x	N.A.
BC174	-	-	-	+	26528	2	23.7	2.9	76.3	30.1	29.0
BC389	-	-	+	+	27074	1	100	2.6	x	x	N.A.
BC52	+	-	-	+	20355	2	67.4	3.8	32.6	29.7	200.0
BC73	-	-	-	-	20583	1	100	1.2	x	x	5.9

+: positive; -: negative; n count: number of counted cells; n pop: number of present cell populations; % min fluo: percentage of cells expressing low or no Gb3 expression; % max fluo: percentage of cells with higher Gb3 expression. N.A.: data not available.

These results show that Gb3 is expressed in epithelial cells of HBCx, and that ESL is useful for the selection of Gb3 positive grafts for future pre-clinical trials.

### Gb3 positive tumor cells can be targeted by STxB conjugate after intravenous injection

In order to study its biodistribution in mice and the possibility to target Gb3 positive tumors, STxB-Cy3 conjugate was injected intravenously. STxB accumulation was studied on fixed tumor and organ specimens by fluorescence microscopy. Gb3 expression (revealed through STxB accumulation) was observed in several organs, including kidney, liver, intestine, and in HBCx. In xenografts, fluorescent signal was observed in tumor cells with epithelial morphology as illustrated in Figure 4E. However, the labeling was heterogeneous in its intensity with uneven intratumoral distribution, including tumor lobules with and without staining. The majority of STxB-positive cells were localized in the periphery of the tumor lobules, whereas central, often necrotic areas were devoid of the staining. Regarding tumor stroma, Gb3 expression was noticed in the majority of cells with endothelial morphology, whereas the rest of stromal cells showed no staining (Figure 4E). Gb3 expression was observed in a variety of organs. Most intense labeling was seen in the kidney, notably in ducts of the medulla (Figure 4C), and with lower intensity in cortical tubules. Glomerular tufts showed low labeling. Furthermore, STxB labeling of low intensity was observed in cells with endothelial morphology of liver (Figure 4D) and in rare epithelial cells of intestinal villa. Lung, spleen and intestine were otherwise STxB negative.

These results demonstrate the possibility of targeting Gb3 expressing HBCx after an intravenous injection, using STxB conjugates. However, non-tumor tissue, notably renal medulla, also shows strong STxB binding.

## Discussion

In the present study, we evaluated Gb3 expression in primary and metastatic human breast carcinomas. Using a novel *ex vivo* STxB labeling (ESL) technique on fine-needle aspirates we observed two groups of primary breast carcinomas (Gb3 positive and Gb3 negative tumors) with different clinicopathological behavior.

There are few reports about Gb3 expression in primary breast cancer [10, 28]. In agreement with a previous study [10], we observed Gb3 expression on the majority of cytological specimens. Gb3 positivity was not correlated with the majority of clinical data, including age, histological type, grade, and mitotic index. We observed a correlation between Gb3 and estrogen receptor expression, which was different from previous data [10]. This apparent disagreement might be due to a low patient number (25 patients) in the previous publication. Several studies reported an increase of Gb3 expression in malignant tumors compared to adenomas or normal tissue [12–14]. Our data on total Gb3 expression levels (using an extraction method) showed that overall Gb3 levels were 1.5 times higher in breast carcinoma versus healthy breast tissue. These results did not reach the significance level, probably because of the low patient number.

The link between Gb3 expression and clinical behavior is still very little explored. Cell differentiation appears to be one of the relevant aspects. Indeed, in pancreatic and ovarian carcinomas a higher level of Gb3 expression was noticed in less differentiated tissue [13, 29]. In agreement with these studies we observed Gb3 expression on cells with malignant cytological characteristics: large tumor cells forming lobular and tubular structures. However, Gb3 was expressed on cohesive epithelial cells and to lower proportion in individualized tumor cells. This observation may indicate that a certain degree of differentiation is needed for Gb3 expression. Supporting this hypothesis, our data show that Gb3 positivity is correlated to estrogen receptor expression. It has been shown that well differentiated human breast carcinomas express higher levels of estrogen receptor [30]. Decrease or loss of Gb3 expression might be associated with malignant behavior as shown for other glycosphingolipids in breast cancer, including GD3, GT3, GQ1bα [31]. Furthermore, reduction of Gb3 was observed in breast cancer cell cultures and was associated with cancer stem cells [32]. However, the clinical relevance of decreased Gb3 expression remains to be addressed. Taken together, Gb3 expression was correlated with estrogen receptor expression and higher morphological cell differentiation, whereas Gb3 negative primary breast tumors were morphologically lower differentiated and showed a statistical link to higher number of lymph node metastases. Our observations indicate that Gb3 positive carcinomas may present lower malignant behavior compared to Gb3 negative tumors.

In the present study, we compared Gb3 expression in primary breast tumors and synchronous lymph node metastases. Half of the patients showed a change in Gb3 expression, including 40% of studied patients with an increase of the relative number of Gb3 expressing cells in metastatic lesions and 10% with decreased expression. Several molecules, including HER2, estrogen and progesterone receptors, show heterogeneous expression between primary tumor and metastases [33]. ER and PR variation is different depending on study and it can reach 7.5% to 35% and 16% to 48.6% for ER and PR respectively. HER2 expression was shown to be more stable with variations between 2.9% to 24% (reviewed in [33]). The causes and mechanisms leading to increase or decrease of these molecules on cancer cells are still unclear. However, several mechanisms were proposed, including cellular differentiation level (i.e. migrating cancer stem cells and epithelial-mesenchymal transition) and accumulation of genetic and epigenetic events during cancer progression [33]. It is probable that similar mechanisms, particularly related to cell differentiation, are implicated in Gb3 expression changes between primary breast cancers and lymph node metastases. Furthermore, we speculate that Gb3 has different functions in both tissue contexts, possibly depending interacting partners. The same molecule could thereby be linked to apparently opposing phenotypes, i.e. a decrease of Gb3 expression with increased metastatic spread in primary tumors, and an increase of Gb3 expression with epithelial tumor cell differentiation in metastatic tissue. This hypothesis is expected to stimulate further research into the molecular mechanisms of glycosphingolipid functions, for which even the most basic aspects are still unexplored.

Discordant expression of molecular markers or target molecules between primary breast cancer and synchronous lymph node metastases is an important issue that can have a significant impact on clinical outcome of the disease and on the choice of appropriate therapy [34]. In the case of STxB-based therapy, enhanced Gb3 expression in primary tumor and metastases might lead to greater therapeutic benefit in patients with highest overall Gb3 expression. Thus, the development of a simple and efficient method for the evaluation of Gb3 expression was needed. For this, we developed the ESL technique for estimation of Gb3 expression on low quantities of freshly obtained tumor cells. It can be applied during routine clinical intervention (i.e. fine-needle aspiration) before chemotherapy or radiation treatment. The heterogeneity of Gb3 expression in breast carcinoma and the relatively low number of epithelial cells that are obtained by one needle pass (ca. 50–100 epithelial cells) might be a limitation of this technique [10], and may require repeated fine-needle aspiration. Other techniques like immunohistochemistry, mass spectrometry, thin layer chromatography, or flow cytometry can be used to quantify Gb3 expression [4, 14, 35]. However these techniques often need expensive equipment, special knowledge, and large amounts of tissue or cells [4].

Increased Gb3 expression in breast cancer and its lymph node metastases indicates that the development of STxB-based therapeutic strategies is of interest in this pathology. STxB-based drugs can target malignant epithelial cells and tumor vasculature as shown for colon and pancreatic cancers [12, 17]. Furthermore, Johansson and colleagues [36] showed recently that cisplatin induced Gb3 expression in malignant mesothelioma cells. Many chemotherapeutic regimens involved in breast cancer treatment include platinum derivatives [37], however the major problem with cisplatin treatment is the development of acquired-drug resistance of the cancer cells [38]. Thus STxB conjugates could be used as second line therapy in treatment of cisplatin resistant breast cancers.

## Conclusion

Despite clinical improvements, major problems are still associated with breast cancer treatment. The development of targeted therapies that effectively eliminate cancer cells with minimal effects on healthy tissue is a major objective in clinical cancer research. The enhanced expression of Gb3 in primary breast carcinoma and their lymph node metastases, as described here, is a favorable condition for the development of STxB-based therapeutic strategies. Furthermore, the newly described ESL technique on fine-needle cytological specimens represents a rapid and cost effective method to determine the Gb3 status of patients prior to making a therapeutic choice.

## Abbreviations

DMEM: Dulbecco’s modified eagle medium
ESL: *Ex vivo* STxB labeling
FNA: Fine needle aspiration
STxB: B-subunit of Shiga toxin.
